# The Diagnosis and Treatment of Infantile Nystagmus Syndrome (INS)

**DOI:** 10.1100/tsw.2006.248

**Published:** 2006-10-30

**Authors:** Sangeeta Khanna, Louis F. Dell'Osso

**Affiliations:** ^1^ Daroff-dell'Osso Ocular Motility Laboratory, Louis Stokes Cleveland Department of Veterans Affairs Medical Center Case Medical School, Cleveland, OH, USA; ^2^ Department of Biomedical Engineering, Case Western Reserve University University Hospitals of Cleveland, Cleveland, OH, USA; ^3^ Department of Neurology Case Western Reserve University University Hospitals of Cleveland, Cleveland, OH, USA

**Keywords:** nystagmus, eye-movement recording, diagnosis, therapy

## Abstract

The successful treatment of infantile nystagmus syndrome (INS) depends primarily on accurate and repeatable diagnosis of the type(s) of nystagmus present as well as their variation with gaze and convergence angles or fixating eye. Research over the past 40 years has demonstrated that the only way to achieve both is by making and analyzing ocular motility recordings. Determination of the direct effects of peripheral and central INS therapies can only be made by pre- and post-therapy comparisons of the nystagmus characteristics, specifically of the quality of the foveation periods within each cycle. If one is only interested in cosmetic improvements, diminution of the nystagmus amplitude is all that need be measured. However, if improvement of visual function is the primary goal of therapy, then measurement of the pre- and post-therapy foveation quality must be made, both in primary position and over a broad range of gaze angles. The use of the eXpanded Nystagmus Acuity Function (NAFX) on nystagmus data yields both an accurate measure of foveation quality and a prediction of maximum potential acuity for the patient's waveform. When used with the patient's measured, pre-therapy visual acuity, the NAFX demonstrates the amount of visual acuity loss that is due to sensory abnormalities, demonstrates the amount due to the nystagmus waveform, and estimates the measured post-therapy acuity for all values of improved NAFX and gaze angles measured. The ability to predict visual acuity improvement was not possible before the use of the NAFX. The failure to incorporate accurate measures of nystagmus waveform and foveation quality into their diagnostic evaluation continues to deprive patients of the best possible standard of care and results in mistaken diagnoses as well as inappropriate and, in some cases, unneeded multiple surgeries.

### INTRODUCTION

#### What is Nystagmus?

Nystagmus is an oscillation of the ocular motor system that begins with a slow eye movement that takes the eyes away from the target or intended gaze angle and is followed by either a return slow eye movement, a saccade, or a combination of both. A saccadic intrusion is initiated by a saccade that takes the eyes away from the target or intended gaze angle and is followed by either a return slow eye movement or a saccade. A saccadic oscillation is a repetitive series of saccadic intrusions.

### Nystagmus in Infancy

There are several types of benign nystagmus usually seen in infancy. The most common types consist of the Infantile Nystagmus Syndrome (INS, aka "congenital" nystagmus — CN), the Fusion Maldevelopment Nystagmus Syndrome (FMNS, aka latent/manifest latent nystagmus — LMLN), the nystagmus blockage syndrome (NBS), and the Spasmus Nutans Syndrome (SNS)[1]. FMNS may be present only with occlusion of one eye (rare) or, more commonly, with both eyes open, but only one fixating. It shares some clinical characteristics with INS, but it is always associated with strabismus and has a directional change with ocular cover, beating towards the fixating eye. NBS is a special case of INS whereby the patient produces a purposive esotropia to either damp the INS or convert it into a low-amplitude FMNS. In both cases, the resulting waveform is more conducive to higher acuity. Spasmus nutans is a dysconjugate pendular oscillation with head nodding and tilt[2]. Because of their different mechanisms and interrelationships with strabismus, INS, FMNS, NBS, and SNS do not respond to the same therapies, making accurate diagnosis a necessity. This review will concentrate on INS and our conclusions and recommendations that have resulted from ocular motility analysis of nystagmus waveforms. In both the diagnosis and treatment of INS, some commonly held clinical impressions have been found to be less than helpful, others simply wrong.

### Infantile Nystagmus Syndrome (INS)

The nystagmus of the INS is an ocular motor disorder that can appear at birth, but is usually noted in early infancy (at the time of development of both motor and visual function) and persists throughout life. INS encompasses both nystagmus that is not associated with ocular anomalies and nystagmus associated with other anomalies (e.g., albinism, aniridia, retinal dystrophies, optic nerve hypoplasia, or congenital cataracts). The syndrome consists of one or more types of nystagmus with characteristic waveforms, head turns, tilts, or oscillations. Rarely, the nystagmus becomes manifest later in life[3], so the term "congenital" should be thought of as a congenital "predisposition" for this particular type of ocular motor instability rather than taken literally. The association of INS with primary visual defects led to the mistaken presumption that the nystagmus was secondary to poor vision and that two different types existed: "sensory defect" and "motor defect". However, eye movement recordings have demonstrated that the specific types of nystagmus found in the INS had the same waveforms and underlying mechanism, regardless of the coincidental, perhaps facilitating, existence of a sensory deficit. The nystagmus itself is the *direct* result of an ocular motor control instability that may develop with or without an accompanying sensory deficit. INS may appear spontaneously or be familial. Hereditary INS may be sex linked, recessive, or dominant; the dominant form has been linked with chromosome 6p12[4]. Recent genetic studies have allowed further identification of genes and genetic loci associated with INS and retinal developmental defects accompanying some forms of INS[5].

Although the waveforms of INS may be pendular or jerk, our eye movement recordings revealed that both stem from the *same* underlying pendular oscillation[6]. Because of that, the originating slow phases of jerk waveforms have increasing velocities with the fast phases being normal saccades bringing the eye back to target. Because of their extremely short foveation times, pure pendular and jerk waveforms are not conducive to good acuity (foveation time is the term used to describe the time intervals when eye position maintains the target image within the foveal area and eye velocity is less than 4°/sec) and, hence, in INS we seldom see pure pendular or jerk, but waveforms with extended foveation. Many of the resulting INS waveforms are distinctive and are not present in acquired nystagmus. These waveforms are an expression of the attempts of the saccadic and fixation subsystems to increase foveation time. Most of the specific waveforms identified in INS are diagnostic, being found in no other type of nystagmus. Individuals and families with INS consistently display the same subset of the 12 known INS waveforms. Unlike INS, FMNS has linear or decreasing velocity slow phases; accurate diagnosis of INS depends on identification of specific waveforms using objective motility recordings. Examples of common INS waveforms are shown in Fig. 1. Although some infants have mature jerk INS waveforms, in others, the waveforms may change with age[7,8,9]. In the early months after onset, the predominant waveforms may be pendular and asymmetric pendular, which by 18 months of age, convert to predominantly jerk waveforms, allowing for better visual acuity through improved foveation. This waveform progression reflects saccadic modification of the INS waveform by the developing visual and motor systems, and may reflect the infant's state of visual attention. Whether or not mature waveforms are present at an early age, eye movement recordings can accurately diagnose infants by detecting those waveforms that are pathognomonic for INS.

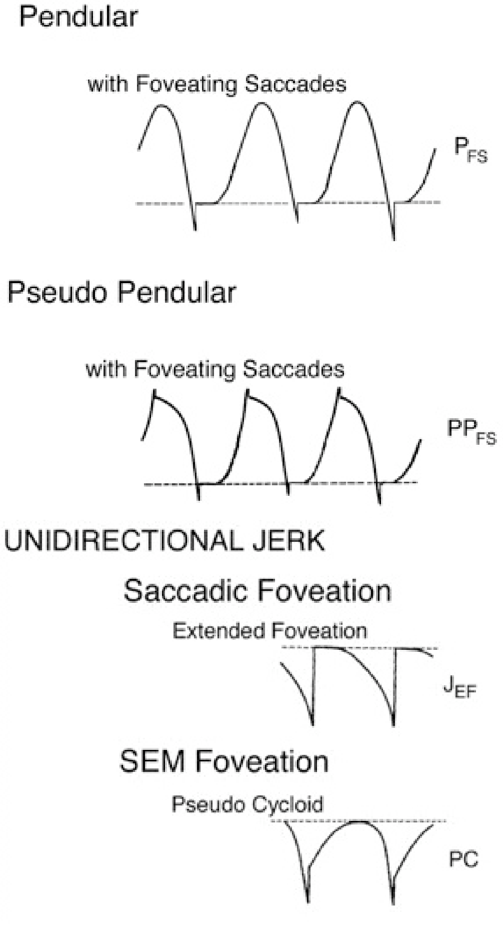

**FIGURE 1.** Common and pathognomonic pendular and unidirectional jerk INS waveforms showing the target position and intervals of extended foveation. Shown are pendular with foveating saccades ($P_{FS}$), pseudopendular with foveating saccades ($PP_{FS}$), jerk with extended foveation ($J_{EF}$), and pseudocycloid (PC).

## CLINICAL CHARACTERISTICS

### Diagnostic Features

The characteristic features of INS are summarized in Table 1. Unfortunately, no combination of clinical features exists that can reliably differentiate INS from FMNS; only eye movement recordings can do so. Because they are necessary for the highest standard of care for nystagmus patients, this paper will presume such recordings are available for both diagnosis and determining the direct effects of treatment.

**TABLE 1**
**Characteristics of Infantile Nystagmus**

| |
|---|
| Binocular with similar amplitude in both eyes |
| Usually horizontal and torsional (vertical rare) |
| Pendular or jerk with increasing velocity slow phases |
| Distinctive waveforms with foveation periods and braking saccades |
| Asymmetric aperiodic alternation possible (Baclofen ineffective) |
| Provoked or increased by fixation attempt |
| Abolished in sleep or inattention to visual tasks |
| Gaze modulated, not gaze evoked |
| Diminished (damped) by gaze-angle or convergence nulls |
| Superimposition of latent component possible |
| Reversal of the IN during optokinetic stimulation (mislabeled, "inversion" of the optokinetic reflex) |
| Associated head oscillation (not compensatory) or turn |
| No oscillopsia except under rare conditions |
| Normal smooth pursuit, optokinetic, and vestibuloocular systems |

### Nystagmus Planes

Although vertical, oblique, and circular waveforms of INS have been reported, the great majority of patients with INS have a mainly horizontal nystagmus, albeit many times with a minor torsional component. One of the striking features of INS is that this horizontal oscillation remains horizontal, even in vertical or oblique directions of gaze, and may not change on occlusion of either eye. This can be an important diagnostic indicator in the absence of eye movement recording. However, some INS patients have a latent component that may cause direction reversal with occlusion; i.e., the INS waveform reverses direction due to a shift in the "null" position. This mimics FMNS clinically, but not when recorded, where the waveforms can be accurately identified as being either INS or FMNS.

### Null Locations and Visual Acuity Fields

An important diagnostic feature of CN is the presence in the majority of patients of a "null" position of gaze ("gaze-angle null") where nystagmus damps. A more accurate characterization is an eXpanded Nystagmus Acuity Function (NAFX) "peak" where the nystagmus waveform is best for acuity (see below for explanation of the NAFX). If the NAFX peak is narrow and not straight ahead (i.e., gaze-angle null), a preferred head turn may appear. Even very young children may adopt this to improve their vision. In this preferred gaze position, the nystagmus waveform has features that facilitate the best possible vision for the patient. Visual function depends on much more than primary position visual acuity, yet that and stereo acuity are all that are usually measured. For patients with INS, the degradation of their acuity with lateral gaze (lateral to their idiosyncratic angle of maximal acuity, aka their "null" angle) may be a more important measure of both visual function and of therapeutic improvement. Therefore, visual acuity should be measured at known gaze angles by fixing the visual stimuli and moving the head to known angles[10]. Another characteristic of INS is that convergence may damp the nystagmus ("vergence null"), allowing near visual acuity to be better than distance acuity; this too, should be measured (see Fig. 2). Convergence also broadens the NAFX vs. gaze angle peak, allowing better acuity over a larger portion of the visual field[11].

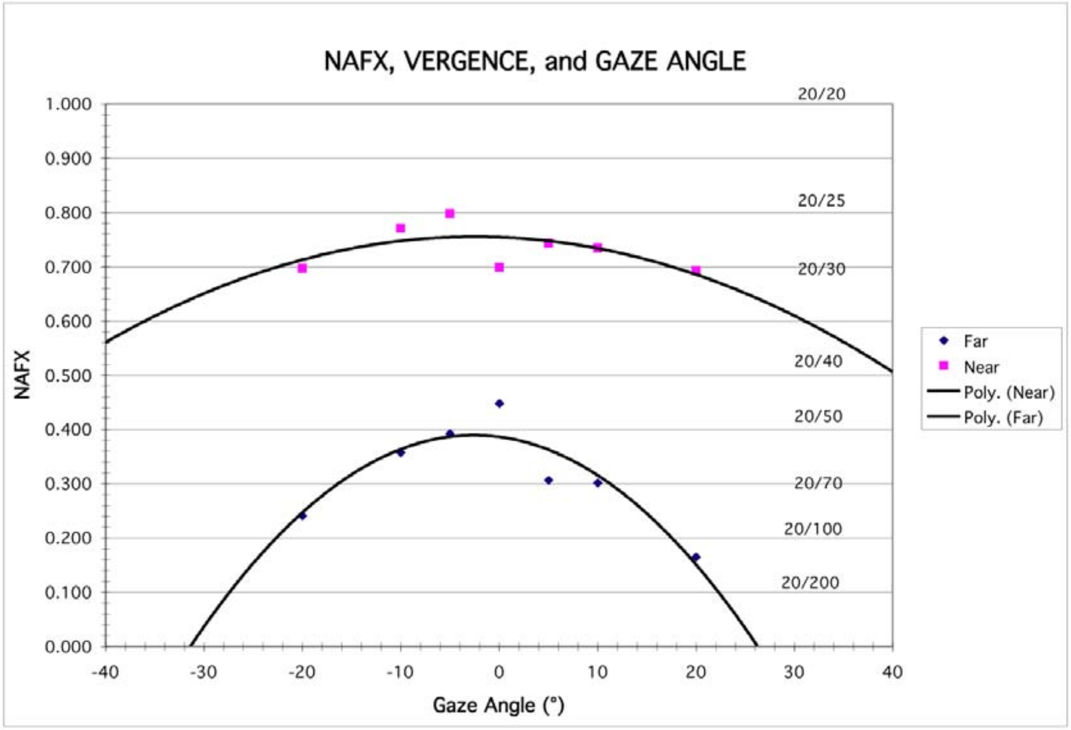

**FIGURE 2.** NAFX variation with gaze angle for far and near targets. Convergence both raises and broadens the peak NAFX curve. Acuities shown are for patients aged 40–60 years old.

### Increase with Visual Effort

The intensity of nystagmus may increase with increasing visual attention ("fixation attempt") and damps with visual inattention and during sleep[12]. Also, the head oscillations that often accompany INS increase with visual intent. Recent evidence has isolated the stress associated with increased fixation attempt as the factor that exacerbates INS rather than the simple act of trying to see higher acuity targets[13].

### Oscillopsia

Individuals with INS usually do not experience an illusory oscillatory movement of their environment (oscillopsia)[14,15]. This lack of oscillopsia in INS, and also in FMNS, suggests that both oscillations occur within an efference copy feedback loop that serves to nullify the effects of retinal image oscillation induced by either of these instabilities[16]. Hence, if a patient presents with a primary complaint of oscillopsia, he or she should be assumed to have an acquired condition until proven otherwise. However, it is not true that oscillopsia never occurs in INS. Indeed, in a large retrospective sample, It has been reported that nearly 40% of patients at least occasionally experienced the perception that their environment was moving[17]. Most of these were associated with exacerbations of their nystagmus by fatigue, illness, stress, or looking in their "nonpreferred" gaze direction.

### Head Nodding

Some INS patients exhibit head nodding, especially when attempting a visually demanding task. We concluded, based on our data and known neuroanatomy, that this reflects the effects of the nystagmus signal that drives the eyes on the neck muscles, to which it has access via the semilunar ganglion[18]. Head nodding is not compensatory since the patient's normal VOR would cancel the effects of head oscillation during the periods of target foveation normally present in the INS waveform. The head tremor in INS can be distinguished from that in acquired disease; it is easily suppressed voluntarily in the former, but not in the latter.

### Associated Ocular Anomalies

As described above, INS may or may not be associated with other ocular anomalies. In a study by Abadi and Bjerre[17], 62% of patients had "idiopathic" INS, which may reflect a selection bias. Other estimates of the prevalence of sensory abnormalities are far higher[19]. Disorders most often associated with INS include albinism and retinal dystrophies, aniridia, achromatopsia, and congenital cataracts. More research is needed to establish whether the nystagmus associated with some of these conditions (e.g., achromatopsia) is INS, some other type of nystagmus, or a combination of the two. Presence of such ocular anomalies separates these children with significant visual impairment secondary to other ocular anomalies from those with only nystagmus. However, it must be stressed here that these INS patients do not differ in the types of nystagmus waveforms; i.e., they all have the same mechanisms driving their nystagmus. Hence, all patients, regardless of the type of nystagmus (jerk or pendular), should have a thorough ocular exam. INS patients do show a higher incidence of astigmatism[17,20] so a cycloplegic refraction should be done in all patients and proper correction advised.

Though these are the general sensory and motor characteristics of INS, there is no single clinical characteristic (or combination of characteristics) that can reliably distinguish INS from other benign forms of nystagmus in infancy (e.g., FMNS) or an acquired neurological nystagmus. For example, the increase in nystagmus and direction change with cover of one eye does not make the diagnosis of FMNS as is commonly stated; it could just as easily be INS with a latent component. Also, INS and FMNS can coexist in patients with strabismus. Eye movement recordings have proved to be the only reliable way of precise diagnosis and management. Thus, in addition to the standard office examination, patients with INS require ocular motility recordings and analysis. The therapies effective in INS may not be effective in FMNS, spasmus nutans, or other acquired nystagmus due to differences in the underlying mechanism of waveforms and interaction with strabismus. Hence, it is crucial to first establish the correct diagnosis by recording and identifying the waveform of the ocular oscillation.

## RECORDING, CALIBRATING, AND ANALYZING INS

Recording and calibrating the eye movements of subjects with nystagmus (or with saccadic intrusions or oscillations) is fundamentally equivalent to recording and calibrating normal eye movements. That is, one needs (1) an accurate recording system, (2) calibration targets at known gaze angles, (3) a means to stabilize the subject's head, (4) real-time monitoring of stimulus and eye movement signals, and (5) a means to verbally instruct and correct the subject. With a little experience, any of the currently available recording systems (infrared reflection (IR), magnetic search coil, or high-speed digital video) is adequate to record horizontal eye movements while the latter two are better suited when both horizontal and vertical eye movements are required. Electro-oculography (EOG) is neither accurate (sensitivity, noise, bandwidth) nor stable (drift) enough for the reliable data needed in ocular motor research. Torsional eye movements require either the search-coil or digital-video methods equipped to sense torsion. Descriptions and specifications of available systems may be found elsewhere. Both IR and digital video are noninvasive and can be used in infants and children; the search coil requires placing a contact lens containing a coil of wire in each eye. The ease of use and accuracy of digital video systems and the relative unimportance of torsional movements to visual acuity makes it the best choice in a pediatric setting.

Accurate recording requires stabilization of the patient's head in addition to one of the above systems. Each eye must be calibrated monocularly (i.e., with the other occluded), establishing both the zero position and gain by using only the foveation periods of the waveforms and simple, nonstress-inducing LED targets, not acuity targets. Properly calibrated records allow measurement of both the phorias behind cover and a dynamic documentation of the variation of phorias and tropias, not appreciated from static clinical determinations. They also make it relatively easy to detect boredom (longer slow phases taking the eyes farther away form the target), distraction (voluntary saccades off target), and changes in the fixating eye (the fixating eye shifts away from the target while the previously deviated eye shifts onto the target). The experimenter can easily detect boredom or distraction in real time and verbally encourage the subject to "look at the target" whereupon the normal waveform will immediately return with the foveating periods on target. Further details of recording and calibrating nystagmus may be found elsewhere[21].

Analysis of the recordings is aimed at evaluation of the waveform at different gaze angles and its foveation quality, an objective measurement of which is the NAFX. It uses the time intervals of foveation periods and their position and velocity SDs to establish a measure of the "quality" of an INS waveform (i.e., how likely it was to allow good acuity). The resulting NAFX vs. acuity line has been made age specific to reflect the variation of visual acuity with age (see Fig. 3, a canine line is also included). The details of using the NAFX software may be found elsewhere[22]. Pre-therapy NAFX values, combined with visual acuity measures, can be used to predict the improvement in both peak NAFX and width of the NAFX peak due to therapy; that translates directly into predicted improvements in peak acuity and the range of gaze angles of high acuity[23].

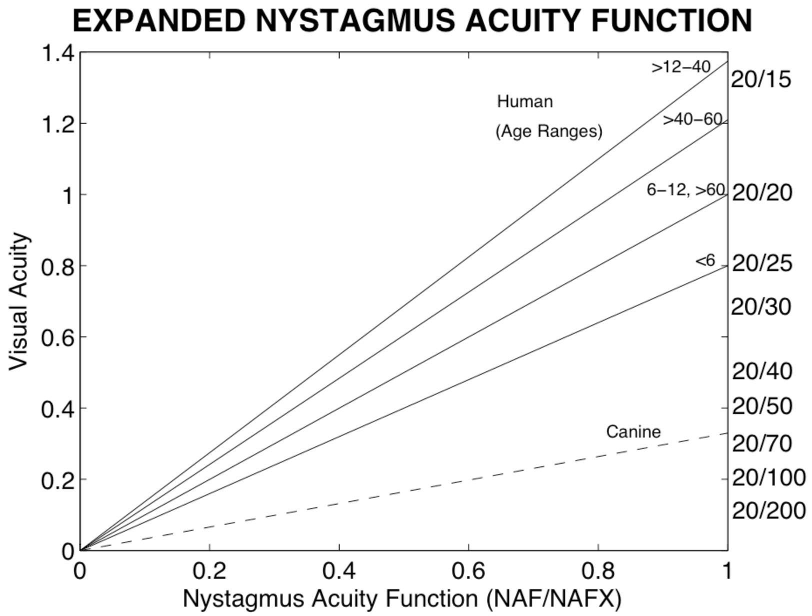

**FIGURE 3.** Relationship between NAFX and Snellen acuity for patients of different ages and for canines.

### THERAPEUTIC APPROACHES TO INS

Current therapies for nystagmus fall into two major categories: *central* and *peripheral*. Central therapies may be neurosurgical or pharmacological; each is directed at the central source of the nystagmus and its aim is to directly reduce the initiating, brain-stem nystagmus signal (the motor command). Peripheral therapies may be pharmacological, optical, or surgical; each is directed at a peripheral mechanism to reduce the resulting eye oscillation directly without affecting the brain-stem nystagmus motor command. One additional, new therapy that may affect nystagmus is *afferent* therapy. An example is gene therapy applied to the retina to correct genetic deficits that impair vision directly and may facilitate the development of nystagmus (e.g., RPE65 deficiency and INS)[24]. Fig. 4 illustrates the anatomical sites of each type of therapy, the neurophysiological signals present, and the measurements of each therapy's direct and indirect effects.

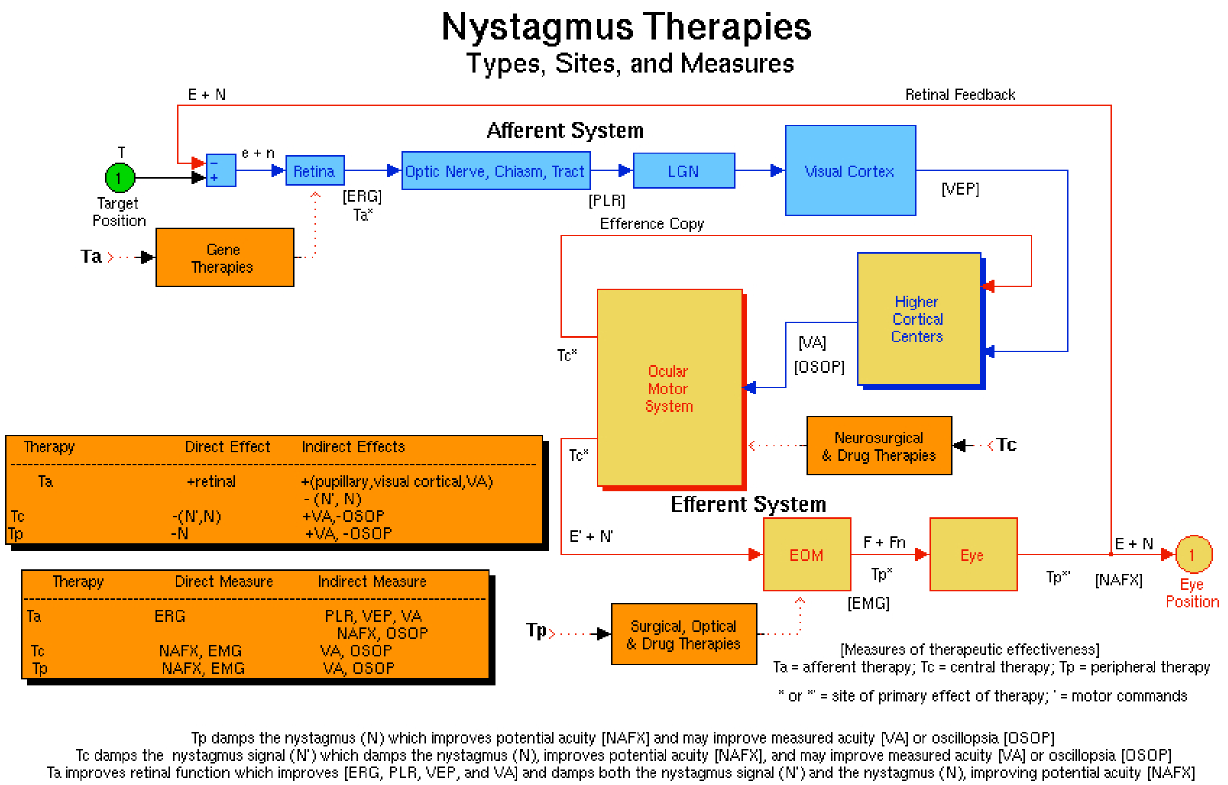

**FIGURE 4.** A block diagram of the ocular motor system indicating the types of therapeutic intervention for nystagmus, their anatomic sites, and direct and indirect measurements of their effectiveness. Ta is afferent therapy for deficits in the visual system (shown here is gene therapy for retinal deficiencies), Tc is central (neurosurgical or drug) therapy for nystagmus, and Tp is peripheral (surgical, optical, or drug) therapy for nystagmus. Shown in square brackets are the sites of therapeutic tests of visual and ocular motor function. The direct and indirect effects of each type of therapy and the direct and indirect outcome measures for each are listed in the Legend Boxes. e = retinal error; n = nystagmus error; E = eye position; N = nystagmus; F = extraocular muscle force; Fn = extraocular muscle nystagmus force; ERG = electroretinogram; PLR = pupillary light reflex; VEP = visual evoked potential; EMG = electromyogram; NAFX = expanded nystagmus acuity function; + = improved, higher, or better; – = diminished, lower, or less; blue/light-blue blocks = afferent sensory; blue/yellow block = afferent-efferent sensorimotor; red/yellow blocks = efferent motor; and black/orange blocks = therapies.

The most accurate measure of any therapy is a measure of its *direct* effects, not an indirect measure of a neurophysiological function that is dependent on other intervening functions, each subject to idiosyncratic deficits. For visual performance measures, before and after therapy in nystagmus, most clinicians only measure resolution limit; visual acuity is the standard for most visual disorders. However, in nystagmus (especially INS), this is neither an accurate nor inclusive measure; the nystagmus may vary with gaze angle and worsen with fixation effort. Also, many patients with INS have one or more afferent visual deficits that limit potential visual acuity, whether or not they were related to the series of events in the motor system that resulted in ocular motor instability. Finally, even for those with no afferent deficits, mental status (stress) often results in a measured visual acuity that is lower than the acuity achieved during normal life when there is no stress. Visual performance in nystagmus is dependent on three factors: retinal image slip velocities, foveation, and the presence ocular and visual pathway anomalies. NAFX analysis of the foveation-period quality for the fixating eye in primary position and the gaze-angle modulation of the NAFX provide direct measures of most therapies and can accurately gauge the improvement of visual function in INS. Therefore, we recommend the use of the NAFX to measure therapeutic effectiveness in nystagmus. When recorded under the same nonstressful conditions used in our lab, the INS waveforms and their measures (e.g., the NAFX) are consistent over time and any changes post-therapy can be ascribed to that therapy.

### Clinical Examination

A complete ocular exam, including slit-lamp examination for iris transillumination and fundus examination, should be performed for other anomalies and should include visual acuity assessment at different gaze angles, head posture assessment, identification of asymmetric (a)periodic alternating nystagmus (APAN), and cycloplegic refraction; APAN often goes undetected, resulting in improper muscle-shifting surgery. Any refractive error should be corrected with glasses or contact lenses (the latter being preferred).

### Eye Movement Recordings

Eye movement recordings give the characteristic waveform of the nystagmus that definitively distinguishes INS from FMNS and other acquired forms of nystagmus based on the distinctive oscillations. That diagnostic information and the patient's idiosyncratic INS variation with both gaze angle and convergence form the backbone for effective therapeutic intervention. The NAFX values over different gaze and convergence angles can be used to predict the expected improvement in peak visual acuity and the range of gaze angles with high acuity as a result of therapy[23]. Different therapies have been tried for INS from prisms to biofeedback including recently reported improvement with gabapentin in a small number of patients[25]. However, the mainstay of therapy remains surgical.

### Surgical Management

Surgical treatment has classically been performed for two main reasons: correction of anomalous head posture and improvement of visual performance. Oscillopsia is not a problem in INS and thus not an indication for treatment, unlike acquired nystagmus. There have been two main types of surgery advocated: (1) Kestenbaum surgery — horizontal recti paired recessions and resections such as to shift the eye position to take advantage of gaze-angle null and (2) artificial divergence surgery — bimedial recessions to create mild divergence (exophoria) of the eyes and take advantage of the convergence-angle null. We recommend the following combinations of surgery based on preop eye movement recordings.

1. If the NAFX improves with convergence and/or convergence damps nystagmus in nonstrabismic, binocular patients, the "artificial divergence"[26] plus tenotomy (see below) surgery is recommended. It consists of bimedial horizontal rectus recessions plus bilateral horizontal rectus tenotomies. The recessions create mild divergence (exophoria) of the eyes such that the patient is forced to employ his fusional vergence to see, and this convergence damps the nystagmus, possibly due to pulley repositioning. This surgery should only be used if the patient has fusion and is usually offered to patients who have responded to a trial with base-out prisms (with –1.0S if prepresbyopic).

2. If there is a sharp NAFX peak and/or compensatory head posture with a "null" zone, the Kestenbaum surgery[27] is the best approach. This is a *four-muscle* surgery consisting of yoke-paired recessions (Anderson)[28] and resections (Goto)[29] of the horizontal recti to shift the resting position of the eyes opposite to an eccentric NAFX peak (i.e., in the direction of the head turn). The two muscles that move the eyes in the direction opposite to the head turn are recessed and the two muscles that move the eyes in the direction of the head turn are resected (see also Lee for a discussion of these surgical techniques[30]). The resulting innervation to primary position places the eyes at the new, centered NAFX peak. Although it was originally usually used only for large head turns, the discovery of important waveform improvements due to the tenotomies that are an integral part of the procedure, now indicate its use for any narrow, eccentric NAFX peak, regardless of the amount of eccentricity or the presence of a head turn.

The same therapeutic benefits may be achieved by a combined Anderson plus tenotomy procedure whereby yoke-paired recessions of two horizontal recti (to shift the resting position of the eyes opposite to an eccentric NAFX peak) plus tenotomies and reattachments to their original insertions of the other pair of horizontal recti (to complete the four-muscle tenotomy damping effect) are performed. Because the four-muscle tenotomy has been identified as the underlying reason for damping INS, two-muscle procedures like the Anderson or Goto should not be used without combining them with tenotomy of the remaining two muscles in the plane of the nystagmus.
3. If both 1 and 2 above apply, the bimedial horizontal rectus recessions plus bilateral horizontal rectus tenotomies procedure is recommended since vergence NAFX peaks are higher than version peaks[31].
4. If there are neither convergence nor gaze-angle improvements in the INS, the relatively new four-muscle rectus tenotomy procedure is recommended[32,33,34,35]. As indicated above, we use the NAFX values to (1) determine which patients should have surgery and (2) predict the expected improvements in both best acuity and the gaze-angle range of high acuity[23]. Only in the rare cases when *both* the pre-therapy NAFX and high-NAFX range are high, would tenotomy be contraindicated; for any other combination, tenotomy will improve one or both of these measures of visual function. Fig. 5 demonstrates the NAFX results of the four-muscle tenotomy procedure on a patient whose initial NAFX was 0.332 (equivalent to a visual acuity of 20/50– = 0.457). The 52.4% improvement of the NAFX to 0.506 corresponded to an increase in acuity to 20/30+ = 0.696.

Tenotomy is thought to affect eye-muscle proprioception and reduce the small-signal (slow-phase) gain to improve nystagmus with or without a "null" zone[36]. Large recessions of all the horizontal recti have been recommended by some strabismus surgeons[37,38] for similar indication with the premise that the force exerted by all the muscle is weakened thereby damping nystagmus. However, this affects the ocular motility and causes undesirable exotropia. Because tenotomy accomplishes INS damping without altering ocular motor homeostasis (i.e., no muscles are moved), we do not recommend maximal recessions of the four horizontal rectus muscles for INS patients, especially those who have binocularity and are at risk to develop diplopia as a result of weakening the eye muscles. Tenotomy's efficacy has been demonstrated in an animal study[33], a masked-data clinical trial[34,35], and subsequently in ~100 INS patients in whom the "null" broadening effects were demonstrated[23]. It has also been used successfully to damp acquired nystagmus and reduce oscillopsia in acquired pendular nystagmus secondary to MS[39] and downbeat nystagmus of unknown etiology[40].

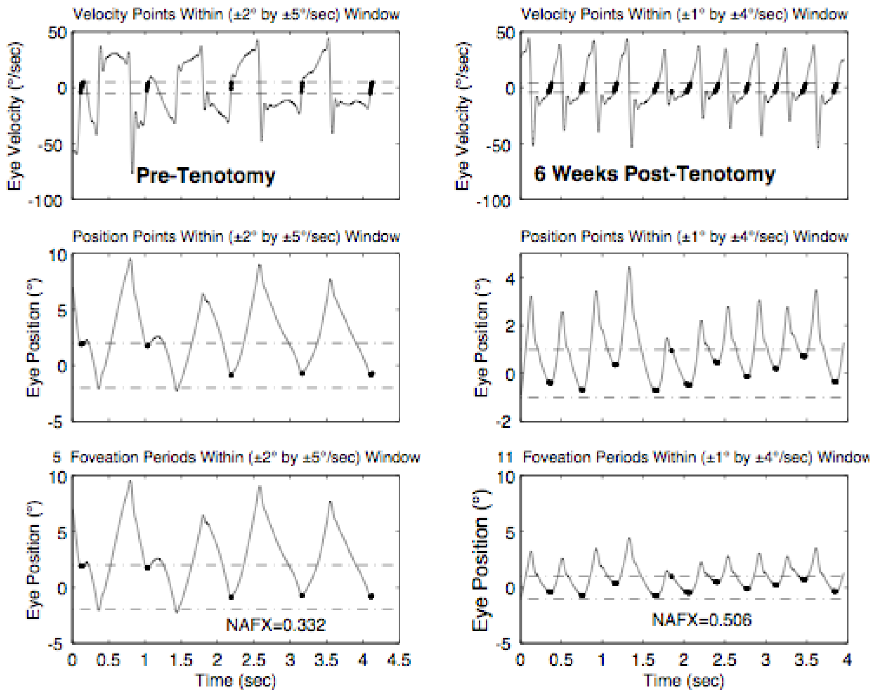

**FIGURE 5.** The output of the NAFX program showing the post-tenotomy improvement over the pretenotomy value. Post-tenotomy, the foveation periods (shown thickened) were more numerous per unit time and of longer duration resulting in a higher NAFX.

INS patients who also have strabismus should have strabismus correction added to either of the surgeries described in 2 or 4 above; convergence damping surgery of INS is contraindicated by strabismus. However, if an esotropia is present, bimedial horizontal rectus recessions may be added to nystagmus surgery to correct the strabismus.

With precise eye movement recording, accurate identification of NAFX peaks allows surgeons to more accurately plan eye muscle surgery without having to rely on the measurement of head posture, which, being under the patient's control, is both variable and unreliable. Postoperative recordings of waveforms have shown the shift in the NAFX peak as predicted and also broadening of the peak area and improvement in the NAFX, thus increasing the visual performance outcome. Multiplanar INS may require two-stage surgeries, first in the plane of the major component of the INS and then, if necessary, in the secondary plane; in some cases, the improvements from the first stage will preclude the necessity for the second.

### Nonsurgical Management

1. Base-out prisms may be used to exploit convergence nulls in binocular INS patients. Usually 7D base-out prisms are added to the patient's refraction with –1.0S added for prepresbyopic patients (the latter must be removed when presbyopia occurs). Once converged, INS remains damped at most gaze angles (especially those in the central ±20° of gaze); therefore, we recommend equal-value, base-out prisms rather than composite prisms for those with both eccentric and near NAFX peaks. This minimizes the amount of prism in each eye and reduces chromatic distortion.
2. For those whose INS damps with afferent stimulation of the ophthalmic division of the trigeminal nerve, soft contact lenses will both correct refractive errors and improve the nystagmus waveform. For other INS patients, contact lenses may also be prescribed for their optical correction and for use in sports. Contact lenses may be prescribed in addition to surgical therapy, providing additional improvement.

### CONCLUSIONS

By employing eye movement recordings and NAFX analysis in the diagnostic workup and therapeutic decision process, accurate and repeatable diagnoses will become the norm and repeat (i.e., corrective) nystagmus surgeries a rarity. Complex cases combining INS, strabismus, latent components, and even FMNS cannot be unraveled nor optimally treated without eye movement recordings and analyses.

### ACKNOWLEDGMENTS

This work was supported in part by the Office of Research and Development, Medical Research Service, Department of Veterans Affairs.
